# First District Dental Society of New York

**Published:** 1886-04

**Authors:** 


### FIRST DISTRICT DENTAL SOCIETY OF NEW YORK.

REPORTED EXPRESSLY FOR THE INDEPENDENT PRACTITIONER.

The regular monthly meeting and clinic of this society was held at the rooms of the S. S. White Dental Manufacturing Company, March 2, 1886.

The attendance at the clinic was reported to be nearly one hundred, and a great interest was manifested in the operations and appliances exhibited. Dr. John J. R. Patrick, of Belleville, Ill., demonstrated his method of making gold crowns for molar and bicuspid teeth, of which he had the dies for forty-eight different sizes and shapes. Dr. Patrick claimed that by his method he can make a crown in ten minutes, and can fit every root perfectly. They are made as follows: The metal is cut out round by cutters, which are used in a small press. Then the cutters are removed, another appliance inserted into the press, by which the flat pieces of metal (in this instance, copper), were pressed into cup-shape. Then the crown dies were put under the press, wherein the metal cups, after they had been annealed, were pressed into the shape of the grinding surface of a molar or bicuspid tooth. This outfit for making artificial crowns is a very perfect one, at the same time saving a great deal of time to the operator.

Dr. H. A. Parr, of New York City, exhibited his new universal separator, applicable in almost every position of the mouth, even in irregularities, although it is not quite free from the objections of an instrument designed for universal application.

Dr. J. M. Crowell, of New York City, showed a platinum plate for continuous gum work, with perforations or small round holes, which had been made with a pair of punching forceps, and the edge of every hole had been compressed in such a manner as to form a complete collar, something like an eyelet. A plate thus prepared is very much stronger than if left smooth, as the body is very firmly held to the plate by the eyelet-like projections of the holes.

Dr. C. F. W. Bödecker alluded to an instance in his own practice, of a patient who, previous to wearing a perforated plate, had broken each continuous gum set in a very few months, but who has worn the perforated plate over a year, and not the slightest check is visible. Dr. Crowell also showed a piece of 18 K gold, with his new gum body flowed upon it. The color was good, and if it will prove serviceable it will be of great benefit to the profession.

Dr. M. Degenhardt, of New York City, exhibited an inferior molar tooth, to the anterior root of which was attached a very large pyogenic membrane.

Dr. E. Parmly Brown, of Flushing, L. I., demonstrated the use of his new moose-hide polishing points. Where applicable, they polish fillings and teeth very quickly and perfectly.

Dr. Brown had promised to set one of his new crowns, which have the platinum pins baked into the porcelain, but he was unable to procure the proper shade.

Messrs. Henry Hertig and H. C Covert exhibited their new gas engine, in connection with Dr. C. F. W. Bödecker's regulator. An examination of this motor has convinced the most of those present that, while at least equally as effective and economical in its consumption of gas for the relative power produced as any other motor, is simpler in the principles of its construction and operation, having no eccentrics, gear wheels, or other parts involving any complication, and it is consequently less liable to derangement, while it works more noiselessly than any motor which has thus far been exhibited.

The motor can be run at a speed of over four hundred revolutions per minute, without the failure of an explosion at each revolution. The cost of running the motor is from one cent to a cent and a quarter per hour, while its power is more than adequate for all dental purposes, being about one quarter of a horse power. It is, moreover, extremely compact, occupying a floor space of less than a square foot, and about two feet in height. The engine can be located, if desired, in the laboratory or cellar below, or in any room above the office, and be easily connected with a lathe, as well as with the suspension engine.

Dr. C. F. W. Bödecker's Regulator consists of a large cone, six inches in diameter at one end and one and one-half inches at the other, which imparts the motion to a transmitting wheel placed on the upper surface of the cone (which upper surface lies horizontal) in such a manner that, while in motion, the transmitting wheel may be moved from the small to the large diameter of the cone, thereby obtaining either the same number of revolutions as pro duced by the motor, four times as many, or any intermediate degree of speed desired. The transmitting wheel, which imparts the motion to the hand piece of the suspension engine, is held a little beyond the smaller end of the cone by a spring, and when in this position it does not revolve. To the bearings of the transmitting wheel a cord is attached, which is brought in connection with the treadle placed near the chair, to be manipulated by the foot of the operator. While the treadle is at rest no motion is transmitted from the cone to the transmitting wheel, but as soon as the treadle is pressed down, the transmitting wheel is pulled forward upon the revolving cone and motion commences. The greater the pressure brought to bear upon the treadle, the nearer the transmitting wheel will approach the base of the cone, and consequently the greater will be the speed, but the moment the foot is removed from the treadle the transmitting wheel is pulled back by the spring beyond the revolving cone, when the revolutions of that wheel, as well as those of the bur in the hand piece, are instantly brought to a dead stop. Another valuable feature of the regulator is the device by which the motion can be instantly reversed. This reverse action is obtained by a pair of wheels playing at right angles upon a wheel situated at the base of the cone, which, at the will of the operator, can be made to revolve alternately either way. By connecting the action of the main shaft to one of the wheels driving the cone, the motion is transmitted in the direction that wheel runs. If the connection is made to the other wheel, the motions of the cone are instantly reversed, which reverse motion is obtained by pressing down a second treadle placed near the operating chair.

When the report of the Clinic Committee had been received, Dr. William Carr, the president, in the chair, called for incidents of office practice.

Dr. H. A. Parr, of New York City, gave a detailed description of his new universal separator, illustrating his remarks by a large sketch of the instrument.

Dr. S. G. Perry, of New York City, was sorry to hear from some gentlemen that, in a few instances, his separators as obtained from the S. S. White Dental Manufacturing Company, did not fit all the teeth they were made for, and expressed the opinion that, if such was the case, the fault was in the manufacturers of the separators. The models, as furnished by him, possessed none of the objections alluded to. Dr. Perry then spoke of the applicability of universal separators. He had found in his experiments that those instruments destined to fit a certain class of tooth only were the most practical. He had, therefore, abandoned the idea of a universal instrument. Dr. Perry then exhibited two new separators invented by his associate, Dr. Woodward.

Dr. C. F. W. Bodecker had used the Perry's separators where applicable, with greatest success, but found that he could never employ them between the lower bicuspids, upper canines and first bicuspids, or very irregular incisors, in which cases he had used Dr. Parr's separator very successfully.

The president then called upon Dr. John J. R. Patrick, of Belleville, Ill., the regular essayist of the evening, to read his paper entitled "The Progressive and Retrogressive Metamorphosis of the Jaws and Teeth." He described the anatomy, formation, and development of the teeth of the entire animal kingdom, and the changes through which they pass. He gave an elaborate description of the mechanism of the jaws of animals, and explained the various modifications of the teeth to conform to the direction and manner in which the forc s are exerted in mastication. The motion of the jaws, he said, corresponds to the character of the teeth implanted in them, and the condyles and muscular arrangements are modified accordingly. Teeth in the human subject were described in the minutest detail as to position, shape, and the marks by which they may be distinguished one from the other, and a delineation given of the changes that take place from the time of the appearance of the deciduous set until the ossification of the pulps of the permanent teeth in old age.

*Dr. Carl Heitzmann*—Congratulated the essayist on the immense amount of study displayed in the elaborate paper, but regretted that he had not applied his energies to giving us something of a new or novel character.

*Dr. Atkinson*—Succeeded him, with his accustomed vigor, in commendation of the speaker of the evening, and the meeting having been protracted until after ten o'clock, the society adjourned.

